# Radiotherapy and AAV-based immunotherapy: A new alliance for cancer treatment

**DOI:** 10.1016/j.omton.2026.201276

**Published:** 2026-06-30

**Authors:** Ainhoa Andueza, Jesús Prieto, Juan Dubrot

**Affiliations:** 1 Solid Tumors Program, Cima-Cancer Center Clínica Universidad de Navarra (CCUN), Pamplona, Spain

## Main text

Radiotherapy (RT) is an essential component of the treatment of many solid tumors; yet, its clinical efficacy is often limited by radioresistance and tumor relapse. It has been shown that the curative potential of RT is enhanced when irradiation-mediated tumor cell killing is accompanied by the activation of antitumor immune responses.^1^ However, RT rarely triggers productive antitumor immunity despite inducing the release of tumor antigens and their presentation to the immune system.^2^ This limitation largely arises because tumor irradiation activates robust immunosuppressive pathways that inhibit both adaptive and innate immunity.^3^

In our recent article published in Cancer Cell, we sought to engineer a gene therapy platform that couples tumor irradiation with the localized expression of immunostimulatory molecules capable of reprogramming the irradiated tumor microenvironment (TME) and driving vigorous antitumor immune responses.^4^ Adeno-associated virus (AAV) vectors meet these criteria as they are versatile platforms for long-term gene expression and have demonstrated a favorable safety profile during years of clinical experience. In addition to that, we found that ionizing radiation (IR) induced epigenetic remodeling of AAV episomes, resulting in enhanced tumor transduction, accentuating its potential as combinatorial treatment.

AAVs are single-stranded DNA vectors that, following cell entrance, deliver their genome to the nucleus, where it undergoes second-strand synthesis and forms circular concatemers that persist extrachromosomally. These episomes associate with histones, giving rise to chromatinized, minichromosome-like structures that are subjected to epigenetic regulation.^5^ We observed that RT induces DNA demethylation and enrichment of H3K27ac at the transgene promoter, changes that are consistent with transcriptional activation.^6^ In addition, RT promotes the binding of YY1 to the 5′ ITR region of the AAV genome. YY1 is a key chromatin organizer and transcriptional regulator that interacts with multiple epigenetic modifiers including histone acetyl transferases.^7^ Mutation of two nucleotides in the YY1-binding site significantly reduces transgene expression, indicating the implication of this factor in the improvement of AAV-mediated tumor transduction after RT. Consistent with the reversible nature of epigenetic modifications, the effect of IR on AAV transgene expression was transient as we observed a progressive reduction in transgene expression when AAV transduction was delayed after irradiation.

For the system to be both safe and effective, transgene expression should be durable and spatially restricted to the irradiated tumor, avoiding off-target payload production. To achieve this, we harnessed the induction of type I interferons (IFNs) by IR^8^ and employed a synthetic IFN-responsive promoter. In this context, RT both triggers and amplifies AAV-mediated transgene expression. To maximize local transgene activity to the target lesion, the vector was administered by local injection into previously irradiated tumors. Consistently, single-photon emission computed tomography/computed tomography (SPECT/CT) imaging in mice receiving AAV with a (Tc-99m)-labeled capsid showed that intratumoral delivery led to preferential accumulation at the injection site, whereas systemic administration resulted in predominant uptake in the liver and spleen. However, even in this scenario, AAV genomes were found in the liver several days after administration, as most AAV serotypes are inherently hepatotropic. Of note, the liver generates low levels of type I IFNs^9^ due to TLR activation by gut-derived bacterial products that could lead to the activation of the AAV-inducible promoter. Hence, we incorporated miR-122 binding sites at the 3′ end of the expression cassette to prevent undesired off-target payload production in hepatocytes.

To demonstrate the versatility of this platform, we designed a panel of monocistronic and bicistronic vectors sharing identical regulatory elements but encoding distinct immunostimulatory cytokines. The vectors were administered intratumorally immediately following irradiation (8 Gy). RT consistently enhanced tumor transduction across all constructs, with each vector shaping the TME according to its encoded payload. Among all tested candidates, the vector encoding singe-chain IL-12 (AAV-iIL12) showed the most potent antitumor activity. IL-12 is a central orchestrator of antitumor immunity, linking innate and adaptive immune responses. It primarily acts through the induction of IFN-γ production by NK and T cells.^10^ Despite promising preclinical efficacy, the clinical development of systemically administered recombinant protein was halted due to elevated serum levels of the cytokine that were associated with unacceptable toxicity. In contrast to systemic IL-12 administration, local delivery of AAV-iIL12 into irradiated tumors allowed for high intratumoral IL-12 concentrations with low serum values, enabling tumor eradication without significant toxicity. This favorable efficacy-tolerability balance stems from precise spatial control of transgene expression conferred by the vector’s regulatory elements.

Local injection of AAV-iIL12 following tumor irradiation elicited tumor regression and prolonged animal survival across a variety of murine subcutaneous and orthotopic tumor models. This potent antitumor activity was associated with marked remodeling of the TME. Treated tumors showed activated ICAM1^+^ endothelial cells together with strong repolarization of the myeloid compartment with decreased immunosuppressive populations and increased frequencies of pro-inflammatory cells expressing elevated levels of the T cell-recruiting chemokines CCL5, CXCL9, and CXCL10. As a result, we detected dense effector/stem-like exhausted CD8^+^ T cell infiltration exhibiting enhanced IFN-γ and TNF-α production. Despite the prominent effect on CD8^+^ T cell recruitment, depletion of this population or deletion of IFN signaling or of MHC class I expression in tumor cells—two common mechanisms of immune evasion—did not restrain the therapeutic efficacy of RT + AAV-iIL12. In contrast, blockade of IFN-γ or FAS-mediated cytotoxicity completely abrogated the antitumor responses, highlighting a critical role for both IFN-γ activity and FAS-FASL interactions in RT + AAV-iIL12-mediated tumor rejection. Overall, the combination of RT and AAV-based delivery of IL-12 represents a promising therapeutic strategy that assembles multifactorial immune mechanisms that can be further reinforced by other clinically relevant immunotherapies such as immune checkpoint inhibitors whose efficacy is closely linked to tumor-infiltrating lymphocyte (TIL) abundance. Importantly and despite the localized intervention, changes induced in the immune system elicited abscopal effects that were further enhanced by concurrent irradiation of distal tumor nodules. These findings suggest that this therapeutic approach may be applicable in the future not only to patients with locally advanced disease but also to those with oligometastatic or widespread metastatic cancer.

**Figure 1:**
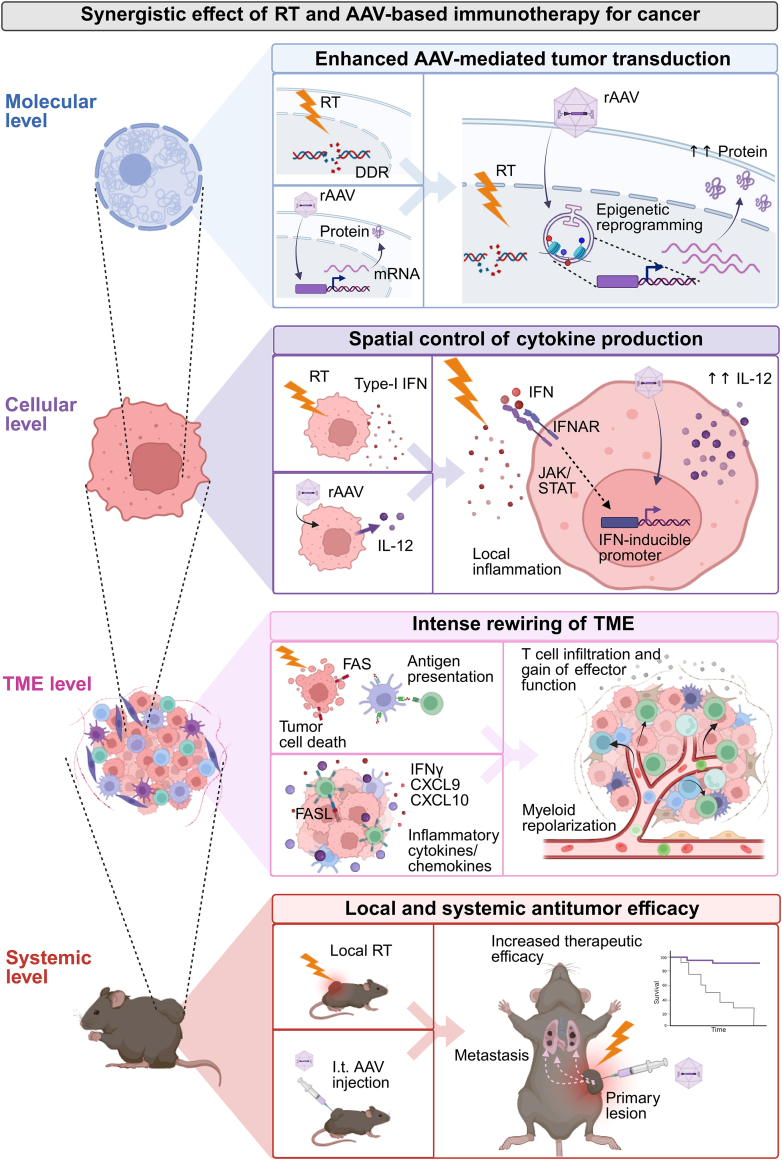
RT and AAV-based immunotherapy interact at different levels to drive potent antitumor immunity At the molecular level, RT induces DNA damage responses (DDRs) and epigenetic reprogramming, which acts on episomal AAV genomes, resulting in enhanced transgene expression. At the cellular level, RT triggers type I IFN production, leading to the activation of the IFN-responsive promoter controlling IL-12 expression and thereby enabling spatially restricted cytokine production within irradiated tissues. At the TME level, RT enhances antigen presentation and upregulates FAS expression on tumor cells, whereas AAV-mediated IL-12 expression induces IFN-γ production and T cell-attracting chemokines. Together, these effects promote myeloid repolarization and the accumulation of activated cytotoxic T lymphocytes expressing FasL within the TME. At the therapeutic level, the combination of RT and intratumoral AAV-iIL12 induces regression of the treated lesion while simultaneously eliciting systemic antitumor immunity with inhibition of distant metastases. Created in https://BioRender.com.

In summary, we have developed an inducible AAV-based immunotherapy platform that engages IR with the activation of antitumor immunity in a clinically applicable manner. This system uniquely exploits multilayered interactions between RT and AAV-based immunotherapy (Figure 1), which can be summarized as follows: (1) RT enhances AAV-mediated tumor transduction through epigenetic reprogramming of the AAV episome; (2) an IFN-responsive promoter restricts transgene expression to irradiated tumors; (3) RT causes tumor cell death and tumor antigen presentation initiating immune responses potentiated by AAV-based cytokine delivery; (4) RT induces FAS expression, thus sensitizing tumors to both antigen-dependent and -independent killing by T and NK cells reinforced by AAV payload; and (4) local therapy with IR and AAV-mediated cytokine expression modulates the immune system to achieve local and systemic antitumor responses.

AAVs have been used clinically for decades to treat a wide range of monogenic diseases; however, their application in oncology has remained limited, in part due to inefficient tumor transduction. We have demonstrated that the combination of RT and AAV-based immunotherapy enhances intratumoral transgene expression and elicits synergistic antitumor effects, positioning AAVs as a versatile new weapon in the fight against cancer.

## Declaration of interests

The authors declare no competing interests.

## References

[1] Weichselbaum R.R., Liang H., Deng L., Fu Y.-X. (2017). Radiotherapy and immunotherapy: a beneficial liaison?. Nat. Rev. Clin. Oncol..

[2] Lussier D.M., Alspach E., Ward J.P., Miceli A.P., Runci D., White J.M., Mpoy C., Arthur C.D., Kohlmiller H.N., Jacks T. (2021). Radiationinduced neoantigens broaden the immunotherapeutic window of cancers with low mutational loads. Proc. Natl. Acad. Sci. USA.

[3] Charpentier M., Spada S., Van Nest S.J., Demaria S. (2022). Radiation therapy-induced remodeling of the tumor immune microenvironment. Semin. Cancer Biol..

[4] Marco S., Fernández M., Honorato B., Juanarena N., Sainz C., Andueza A., Gould D.J., Anderson S., de Andrea C., Domínguez P.P. (2026). Radiotherapy synergizes with an inducible AAV-based immunotherapy platform to program local and systemic antitumor immunity. Cancer Cell.

[5] Gonzalez-Sandoval A., Pekrun K., Tsuji S., Zhang F., Hung K.L., Chang H.Y., Kay M.A. (2023). The AAV capsid can influence the epigenetic marking of rAAV delivered episomal genomes in a species dependent manner. Nat. Commun..

[6] Pekrun K., Stephens C.J., Gonzalez-Sandoval A., Goswami A., Zhang F., Tarantal A.F., Blouse G., Kay M.A. (2024). Correlation of antigen expression with epigenetic modifications after rAAV delivery of a human factor IX variant in mice and rhesus macaques. Mol. Ther..

[7] Dong X., Guo R., Ji T., Zhang J., Xu J., Li Y., Sheng Y., Wang Y., Fang K., Wen Y. (2022). YY1 safeguard multidimensional epigenetic landscape associated with extended pluripotency. Nucleic Acids Res..

[8] Burnette B.C., Liang H., Lee Y., Chlewicki L., Khodarev N.N., Weichselbaum R.R., Fu Y.-X., Auh S.L. (2011). The efficacy of radiotherapy relies upon induction of type i interferon-dependent innate and adaptive immunity. Cancer Res..

[9] Wu M.-S., Kuo Y.-P., Lo Y.-C., Tsai D.-J., Lai C.-Y., Chuang T.-H., Lin S.-Y., Tsai W.-T., Chung P.-J., Yu G.-Y. (2021). Type I interferon signaling accelerates liver regeneration by metabolic modulation in noninfectious conditions. Am. J. Pathol..

[10] Xu Y., Sun X., Tong Y. (2024). Interleukin-12 in multimodal tumor therapies for induction of anti-tumor immunity. Discov. Oncol..

